# Quantifying test-retest reliability of repeated objective attentional measures in Lewy body dementia

**DOI:** 10.1007/s00415-022-10977-4

**Published:** 2022-01-27

**Authors:** Greg J. Elder, Sean J. Colloby, Michael J. Firbank, John-Paul Taylor

**Affiliations:** 1 Northumbria Sleep Research, Department of Psychology, Faculty of Health and Life Sciences, Northumbria University, Newcastle upon Tyne, NE1 8ST UK; 2 Translational and Clinical Research Institute, Newcastle University, Campus for Ageing and Vitality, Newcastle upon Tyne, NE4 5PL UK

**Keywords:** Lewy body dementia, Attention, Reaction time, Trials, Outcome measures

## Abstract

Objective cognitive impairment is a feature of Lewy body dementia (LBD), and computerised attentional tasks are commonly used as outcome measures in interventional trials. However, the reliability of these measures, in the absence of interventions, are unknown. This study examined the reliability of these attentional measures at short-term and longer-term follow-up stages. LBD patients (*n* = 36) completed computerised attentional tasks [simple and choice reaction time, and digit vigilance (SRT, CRT, DV)] at short-term (Day 0–Day 5) and longer-term (4 and 12 weeks) follow-up. Intra-class correlations (ICCs) were calculated to assess test–retest reliability. At short-term, the reciprocal SRT, CRT and DV mean reaction time to correct answers, the reciprocal DV coefficient of variation, and reciprocal power of attention (PoA) all showed excellent levels of reliability (all ICCs > 0.90). The reciprocal PoA showed the highest level of reliability (ICC = 0.978). At longer-term follow-up, only the reciprocal PoA had excellent levels of reliability (ICC = 0.927). Reciprocal SRT, CRT and DV reaction time to correct answers, and the CRT coefficient of variation values, showed good levels of test–retest reliability (ICCs ≥ 0.85). Contrary to expectations, most attentional measures demonstrated high levels of test–retest reliability at both short-term and longer-term follow-up time points. The reciprocal PoA composite measure demonstrated excellent levels of test–retest reliability, both in the short-term and long-term. This indicates that objective attentional tasks are suitable outcome measures in LBD studies and that the composite PoA measure may offer the highest levels of reliability.

**Supplementary Information:**

The online version contains supplementary material available at 10.1007/s00415-022-10977-4.

## Background

Lewy body dementia (LBD) is an umbrella term that refers to dementia with Lewy bodies (DLB) and Parkinson’s disease dementia (PDD). DLB is a common cause of neurodegenerative dementia in older people and accounts for as much as 7.5% of diagnosed dementia cases in secondary care [1, 2]. Dementia is frequently observed as a long-term outcome in Parkinson’s disease (PD), since up to 80% of individuals with PD eventually develop dementia [3, 4]. DLB and PDD share a common underlying alpha-synuclein neuropathology, and both dementias share the core diagnostic and clinical features of marked attentional and visuoperceptual deficits [5, 6].

Clinically, patients with LBD report impairments in subjective cognition. A common symptom is that of cognitive fluctuations, which refers to interruptions in awareness, reductions in alertness and transient episodes of confusion [7]. Objective impairments in cognition, measured using computerised reaction time tasks, are also apparent in LBD. Individuals with LBD display similar levels of objective attentional impairment, where this is more pronounced than is observed in Alzheimer’s dementia (AD) [8, 9]. Compared to patients with AD, those with LBD display slower reaction times (RTs) and higher levels of intra-individual variability [7, 10–12]. Attentional dysfunction can negatively affect quality of life in PDD [13] and may also contribute to other frequently-observed symptoms, including visual hallucinations [5, 14].

Objective measures of attention are frequently used as primary or secondary outcome measures in pharmacological and non-pharmacological LBD clinical trials [15–17]. This is also the case in ongoing, or planned, clinical trials, where objective attentional measures such as psychomotor speed, attention and memory, or composite objective measures (e.g. Continuity of Attention) are considered to be primary outcome measures (e.g. ClinicalTrials.gov; NCT03305809; NCT04739423). However, it is not currently known if measures of objective attention are stable when they are repeated at later time points, either in the short term, or over longer time spans, and it is important to understand this given the potential variance introduced by cognitive fluctuations. There is therefore a clear need to determine the stability of these outcome measures to validate their use and to aid the interpretation of these variables within the context of clinical trials [2, 18]. This is particularly important when objective attentional or reaction time-based measures are assessed as primary outcomes.

The aim of the present study was to assess the test–retest reliability of objective attentional outcome measures in LBD between baseline and five consecutive days (short-term follow-up), and between baseline and follow-up time points of 4 weeks and 12 weeks (longer-term follow-up). These time points are typical of those used in LBD clinical trials. The present study examined mean reaction time and the intra-individual variability in reaction times, as an objective marker of attentional fluctuations. This study also aimed to estimate the minimum number of trials needed to achieve acceptable levels of test–retest reliability, in order to potentially reduce task duration and intensity for future studies. Finally, this study also aimed to inform indicative sample sizes for LBD clinical trials where these measures are desired to be used as primary outcome measures.

It was hypothesised that individuals with LBD would demonstrate poor test–retest reliability in 1) overall reaction times; 2) intra-individual reaction time variability; 3) composite measures of focussed attention and central information processing speed. It was expected that this would be the case in the short-term and at longer-term follow-up time points.

## Methods

### Participants

Study participants were originally recruited to a randomised controlled clinical trial for an intervention to treat visual hallucinations (ISRCTN40214749) and therefore, all participants reported having visual hallucinations of a moderate-to-severe nature prior to study entry. All data were obtained from study procedures conducted during the clinical trial. Data were collected between November 2013 and December 2017. A total of 40 participants (DLB *n* = 26, PDD *n* = 14; *M*_age_ = 75.52 years; SD_age_ = 8.69 years) were originally entered into the study and four participants dropped out of the study prior to the treatment week. Participants met diagnostic criteria for probable DLB or PDD [2, 5], as verified by two experienced clinicians.

Full inclusion and exclusion criteria, recruitment details and participant characteristics from this trial are reported in full elsewhere [19], but briefly, participants were recruited from clinical services in the North East of England and were ≥ 60 years of age. Participants were enrolled in the study if they had no changes to relevant medication (e.g. anti-parkinsonian or psychotropic medication, or cholinesterase inhibitors) in the month preceding participation, had a Mini-Mental State Examination [MMSE; 20] score of ≥ 12, and a sufficient level of English to allow for participation. Exclusion criteria included a history of excess alcohol intake, concurrent major psychiatric illness, and concurrent significant physical illness, co-morbidities or neurological disorders. All participants and their informants (participant carers/relatives) provided written informed consent and the study was approved by an NHS Research Ethics Committee (REC reference: 13/YH/0292).

### Control participant group

Computerised task data were obtained from an additional group of 26 healthy control participants as a non-dementia comparison group (15 male, 11 female; M_age_ = 74.38 years; SD_age_ = 7.22 years). These data were taken from two separate LBD observational studies, where participants were specifically recruited as comparator groups to dementia patients. All participants were recruited separately and provided written informed consent, including for the re-use of their anonymised data. Both studies were approved by an NHS Research Ethics Committee (REC references: 13/NE/0252; 13/NE/0359).

### Measures

Part III of the Parkinson’s Disease Rating Sale was used to assess motor function [21]. Global cognitive function was assessed using the MMSE [20] and Cambridge Cognitive Examination [CAMCOG; 22]. Memory and executive function domain subscores were also derived from the CAMCOG. Informants provided subjective ratings of the presence and severity of participant cognitive fluctuations using the One Day Fluctuation Scale (ODFAS) and Clinical Assessment of Fluctuation (CAF) scale [23], which assess cognitive fluctuations over the previous day and month respectively. Only the LBD patient group completed these measures.

### Computerised attentional tasks

Participants completed three attentional computerised tasks in the current study: simple reaction time (SRT), choice reaction time (CRT) and digit vigilance (DV) tasks, which have been previously shown to be differentially sensitive to LBD, relative to healthy control individuals and other dementias [24]. Tasks were programmed in MATLAB (Mathworks Inc) using the Cogent toolbox (www.vislab.ucl.ac.uk/cogent_2000.php). A laptop PC was used to present the tasks and participants responded using custom buttons held in the dominant hand (SRT, DV), or in both hands (CRT), depending on the task.

In the SRT task, a target (letter X) was displayed for a maximum of 3000 ms per trial, with a varying inter-stimulus interval, and participants were required to respond to the target as quickly as possible. In the CRT task, a target arrow which pointed left or right was displayed for a maximum of 3000 ms and participants were required to respond using the corresponding button. During the DV task, a target (number 9) was continuously displayed on the right of the computer screen, and a series of numerical digits were randomly displayed in the centre of the screen at 500 ms intervals. Participants were required to press a button whenever the centre digit and the target digit matched. The SRT and CRT had 30 trials, and the DV task had 360 trials, where 36 of those were target responses.

### Procedure

All study procedures were completed in the participant’s usual residence (a home or a residential care home environment) on Day 0 and from Day 2 to Day 4. Procedures were completed within a clinical research environment on Day 1 and Day 5 due to the requirements of the clinical trial, unless the participant could not travel, in which case they were assessed in their usual residence. Both follow-up assessments (4 and 12 weeks) were completed in the participant’s usual residence.

After providing informed consent, participants completed demographic and clinical measures at baseline, which were completed with the participant’s informant. Computerised attentional measures were repeated daily, at the same time of day wherever possible, between Day 1 and Day 5. The MMSE, CAMCOG, CAF and ODFAS were repeated at all follow-up stages (Day 5, 4 weeks and 12 weeks). For control participants, only baseline (Day 0) computerised attentional task data were used.

### Data Analysis

Complete data were provided by a total of 36 LBD participants at baseline (23 DLB, 13 PDD), complete follow-up data were obtained from 36 participants at Day 5, from 30 participants at 4 weeks follow-up, and from 29 participants at 12 weeks follow-up. Comparator task data were obtained from 26 control participants.

Attentional task outcome measures (SRT, CRT and DV) included the percentage of correct answers, the mean reaction time (RT) to correct answers, expressed in milliseconds (ms), and the coefficient of variation (COV) of mean RTs to correct answers. The COV is a marker of intra-individual variability and was calculated as (SD_RT_/*M*_RT_) × 100. Two additional composite measures were calculated: firstly, the Power of Attention (PoA), which is a measure of focused attention and psychomotor/information processing speed [15], was calculated by summing SRT, CRT and DV reaction times to correct answers (ms), and secondly, the Cognitive Reaction Time (CogRT), which provides a measure of central information processing speed [15] was calculated by subtracting SRT mean RTs to correct answers from CRT mean RTs to correct answers (ms).

### Attentional task comparison with healthy controls

Untransformed attentional task data (SRT, CRT, DV, PoA and CogRT) were compared between LBD patients and controls using Mann–Whitney *U* tests, with *p* values adjusted for multiple comparisons (adjusted *p* value = 0.005).

### Test–retest reliability

The test–retest reliability of attentional variables (SRT, CRT and DV % of correct answers; SRT, CRT and DV mean reaction times to correct answers; SRT, CRT and DV coefficients of variation (COV); PoA and CogRT) was examined between baseline and Day 1 to Day 5 (short-term reliability) and between baseline, Day 5, 4 weeks and 12 weeks, to examine follow-up reliability. Only participants with complete data were included in analyses. To examine LBD test–retest reliability, all attentional variables, with the exception of the percentage of correct responses, and CogRT, were transformed using the reciprocal (1/RT) due to the non-normality of the data.

For each attentional variable, the test–retest reliability was assessed using an intra-class correlation coefficient (ICC). ICCs were calculated using a two-way mixed model with absolute agreement, based on the mean of multiple measurements [model ICC (A,1); 25]. ICC values ranged from 0 to 1, where low ICC values represent low levels of test–retest reliability, and high ICC values represent high levels of test–retest reliability. This was done separately for baseline and Day 1–5 data, and for baseline, 4 week and 12 week follow-up data. ICC values and 95% confidence intervals (95% CIs) are reported for each attentional variable. Specifically, in the present study, ICC values of < 0.50 were considered to represent poor reliability; values of 0.50–0.75 represented moderate reliability; values of 0.75–0.90 indicated good reliability, and values of ≥ 0.90 represented excellent reliability [26]. The test–retest reliability of participant cognitive measures (MMSE, CAMCOG total and CAMCOG subscores, CAF and ODFAS) was also examined in the same manner between Day 0–5, and between Day 0, 4 weeks and 12 weeks.

### Additional reliability analyses

Three additional reliability analyses were conducted. Firstly, the test–retest reliability of a reduced number of trials was examined. This was done to estimate the minimum number of trials which might be needed to obtain acceptable levels of test–retest reliability whilst minimising participant burden. To examine this, ICCs (two-way mixed models with absolute agreement (A,1)) were calculated for the reciprocal mean RT to correct answers for the CRT task, representing the task with the highest levels of test–retest reliability. This was done using blocks of 10 (ICC10) and 20 (ICC20) trials.

Secondly, to provide target sample sizes for subsequent interventional studies where objective attentional measures are desired as the primary outcome, RT measurements at baseline and 12 weeks were used to determine the standard deviation for the change in reciprocal RT SD_diff_ between those time points. This SD_diff_ was used to calculate effect sizes for clinically relevant changes in RT. For a mean reaction time RT and a change of Δ*T*, the change in reciprocal RT is given by 1/(RT − Δ*T*) − 1/RT. We used the pwr package (version 1.3.0 https://cran.r-project.org/web/packages/pwr) in *R* (R Core Team, 2019) to calculate sample size with a target power of 80% and significance level of 0.05. This was done for CRT and PoA.

Finally, to explore whether reliability might be influenced by the severity of cognitive fluctuations, participants were divided into “low” (*n* = 12) and “high” fluctuator (*n* = 24) groups. This was done on the basis of CAF scores, where a CAF score ≤ 5 was considered low and a CAF score of 6 and above was considered high, with the presence of clinically-significant fluctuations [27]. ICC values were calculated between Day 0–5.

## Results

Baseline participant demographic and cognitive measures are summarised in Table 1.

**Table 1:** LBD patient baseline demographic and cognitive data (*n* = 36)

	Mean	SD
Age (years)	75.16	7.96
Gender (male/female)	27 (75%) / 9 (25%)	
DLB/PDD	23 (63.9%) / 13 (36.1%)	
UPDRS-III^a^	34.66	23.37
MMSE	18.03	6.24
CAMCOG (total)	59.19	21.91
CAMCOG (memory subscale)	13.36	6.42
CAMCOG (executive function subscale)	9.03	4.14
CAF	2.81	2.97
ODFAS	3.16	3.24

^a^*n* = 35

### Comparison of data to healthy control participants

Relative to the control group, LBD patients demonstrated significant impairments in all objective attentional variables (all *p* values < 0.005; Table 2).

**Table 2:** Control and Lewy body dementia patient Day 0 attentional task comparisons

	Control (*n* = 26)		LBD (*n* = 36)	
	Mean	SD	Mean	SD
SRT: correct answers (%)	100.00	0.00	85.88	23.14^a,^***
SRT: mean RT (ms), correct answers	339.57	59.97	888.66	776.45^a,^***
SRT: coefficient of variation (%)	20.72	11.29	63.96	36.65^a,^***
CRT: correct answers (%)	97.05	6.49	68.80	33.76***
CRT: mean RT (ms), correct answers	526.42	81.62	1204.13	1163.88^a,^***
CRT: coefficient of variation (%)	18.02	6.93	58.12	37.55^a,^***
DV: correct answers (%)	96.58	9.76	58.95	25.83***
DV: mean RT (ms), correct answers	516.39	52.74	717.97	155.00***
DV: coefficient of variation (%)	13.17	3.66	33.92	17.20***
PoA	1382.38	143.80	2818.27	1693.47^b,^***
CogRT	186.85	59.06	355.38	1192.64^b,^***

*SD* standard deviation, *SRT* simple reaction time, *RT* reaction time, *CRT* choice reaction time, *DV* digit vigilance

^a^*n* = 34; due to incomplete data

^b^*n* = 32; due to incomplete data

^***^p < .001 (control vs. LBD)

### Short-term reliability

The test–retest reliability of short-term attentional measures, assessed between baseline and between Day 0 and Day 5, was excellent (ICCs > 0.90) for the reciprocal SRT (ICC = 0.924), CRT (ICC = 0.970) and DV (ICC = 0.951) mean RT to correct answers, as well as the reciprocal DV coefficient of variation (ICC = 0.973) and reciprocal PoA, which showed the highest level of reliability (ICC = 0.978). Reciprocal CRT COV% values showed good levels of reliability (ICC = 0.816) and reciprocal SRT COV% showed moderate levels of reliability (ICC = 0.651). Finally, the test–retest reliability of the CogRT was poor (ICC = 0.434). These are summarised in Table 3. When the stability of these measures were examined in the placebo group alone, the resulting ICC values were similar (data not shown). With the exception of the CogRT measure, low and high cognitive fluctuation groups displayed comparable levels of short-term test–retest reliability (Supplementary Table 1).

**Table 3:** Test–retest reliability of Day 0 to Day 5 attentional measures

Attentional measure	ICC	95% CI
SRT: correct answers (%)^a^	0.692	(0.491–0.833)
SRT: 1/mean RT to correct answers^a^	0.924	(0.873–0.959)
SRT: 1/coefficient of variation (%)^a^	0.651	(0.419–0.811)
CRT: correct answers (%)^b^	0.770	(0.487–0.893)
CRT: 1/mean RT to correct answers^a^	0.970	(0.950–0.984)
CRT: 1/coefficient of variation (%)^a^	0.819	(0.701–0.902)
DV: correct answers (%)^a^	0.898	(0.830–0.945)
DV: 1/mean RT, correct answers^c^	0.951	(0.918–0.974)
DV: 1/coefficient of variation (%)^d^	0.973	(0.652–0.890)
1/PoA^e^	0.978	(0.963–0.989)
CogRT^d^	0.434	(0.042–0.702)

^a^*n* = 31

^b^*n* = 33

^c^*n* = 30

^d^*n* = 29

^e^*n* = 27

### Follow-up reliability

Reciprocal composite PoA measure demonstrated excellent (ICC > 0.90) levels of test–retest reliability between baseline and follow-up time points (ICC = 0.927; Table 4). Reciprocal SRT, CRT and DV reaction time to correct answers, and the CRT coefficient of variation values, showed good levels of test–retest reliability (ICCs ≥ 0.85) whereas reciprocal SRT and DV coefficient of variations, and the composite CogRT measure, all displayed poor levels of test–retest reliability (all ICCs < 0.32).

**Table 4:** Test–retest reliability of Day 0 and follow-up (Day 5, 4 and 12 weeks) attentional measures

Attentional measure	ICC	95% CI
SRT: correct answers (%)^a^	0.895	(0.805–0.950)
SRT: 1/mean RT to correct answers^b^	0.849	(0.700–0.931)
SRT: 1/coefficient of variation (%)^c^	0.277	(− 0.481–0.674)
CRT: correct answers (%)^d^	0.896	(0.810–0.950)
CRT: 1/mean RT to correct answers^c^	0.893	(0.780–0.953)
CRT: 1/coefficient of variation (%)^b^	0.874	(0.743–0.945)
DV: correct answers (%)^b^	0.843	(0.706–0.926)
DV: 1/mean RT, correct answers^c^	0.766	(0.539–0.892)
DV: 1/coefficient of variation (%)^b^	0.159	(− 0.607–0.606)
1/PoA^e^	0.927	(0.846–0.969)
CogRT^e^	0.314	(− 0.459–0.709)

^a^*n* = 24

^b^*n* = 23

^c^*n* = 21

^d^*n* = 25

^e^*n* = 20

### Test–retest reliability of cognitive measures

Short-term levels of test–retest reliability were poor for the CAF (ICC = 0.223); moderate for the ODFAS (ICC = 0.543), good for CAMCOG memory and executive subscales (ICCs = 0.884 and 0.846) and excellent for the MMSE and CAMCOG total (ICCs = 0.932 & 0.938; Supplementary Table 2). Between baseline, and 4-week and 12-week follow-up time points, the MMSE, CAMCOG total, CAMCOG memory and CAMCOG executive function subscales all displayed excellent test–retest reliability levels (all ICCs ≥ 0.90), and the CAF and ODFAS both displayed good reliability levels (ICCs = 0.818 and 0.719 respectively; Supplementary Table 3).

### Minimum number of trials

ICC values, calculated on the basis of a shortened version of the CRT between Day 0 and Day 5, showed excellent levels of reliability for both 10 and 20 individual trials for the reciprocal mean RT to correct answers: ICC10 = 0.945 (95% CI 0.907–0.972); ICC20 = 0.965 (95% CI 0.941–0.982).

### Target sample sizes

Based on a hypothetical within-participants study where attentional measures were the primary outcome measure, the SD on the difference in score between Day 0 and 12 weeks was 3.8 × 10^–4^ s^−1^ for CRT and 9.3 × 10^–5^ s^−1^ for PoA. To observe a change in CRT from 1204 ms (Table 1) over 12 weeks, the estimated required sample size at 80% power for a change of 100 ms is *n* = 200; 200 ms would require *n* = 43, and for a change of 300 ms, *n* = 17. For PoA, with a mean baseline of 2818 ms, the required sample size for a change of 200, 400, or 600 ms would be *n* = 95, *n* = 22 or *n* = 10 respectively.

## Discussion

Contrary to expectations, there were high levels of test–retest reliability for some, but not all, attentional measures. This was the case at short-term and follow-up time points. Specifically, overall reaction times and intra-individual reaction time variability displayed high test–retest reliability in the short-term (Baseline and Day 1–5) and between baseline and follow-up time points (4 and 12 weeks). The composite Power of Attention measure showed high test–retest reliability, but cognitive reaction time, as a marker of central information processing speed, showed a poor test–retest reliability. These findings are not in line with the hypotheses that individuals would demonstrate poor test–retest reliability in: (1) overall reaction times; (2) intra-individual reaction time variability and (3) composite measures of focussed attention and central information processing speed, at both short-term and longer-term follow-up. Potential reasons for this include the possibility that whilst objectively, patients with LBD demonstrate high levels of intra-individual variability, participants may have had the same relative degree of objective attentional instability, resulting in a minimal impact upon attentional measures [10–12]. Finally, all patients were taking medications including cholinesterase inhibitors, which can affect attention [15]. However, medication withdrawal prior to study entry was not feasible given the trial design and the potential for the clinical deterioration of participants. Overall, these results still indicate that in the short-term, attentional measures which are commonly used in LBD clinical trials, generally display very high levels of test–retest reliability, even at 12 weeks follow-up. These findings have important implications for the design and delivery of LBD clinical trials using these measures as outcomes, as our study suggests that many of these are reliable.

These findings indicate that the reciprocal Power of Attention composite measure displayed excellent levels of reliability, and appears to be a very robust marker of cognition in LBD, even at follow-up time points of up to 12 weeks. Although a period of 12 weeks is in line with previous pharmacological clinical trials in LBD, and the findings of the present study are relevant to trial design [28, 29], future studies should specifically examine whether these high reliability levels persist beyond 12 weeks. This is important as within clinical trials, it is common to assess patients over a longer period of time; for example, LBD trials report follow-up time points of approximately 6 months [15, 30] or 1 year [31]. Subjective declines in cognition, with a large degree of variation, have been observed over longer time periods in LBD [32] and this might also be the case with objective measures. Another important finding in the present study was that objective attentional measures displayed comparable levels of reliability to the MMSE and CAMCOG. Given the ease and standardisation of objective test administration, these may be suitable trial outcome measures.

In terms of subjective measures, the poor short-term reliability of the CAF may reflect its intended use as a measurement tool for cognitive fluctuations in the preceding 4 weeks [23]. However, the ODFAS demonstrated moderate short-term reliability, and good longer-term reliability, which is surprising as this should only measure cognitive fluctuations in the preceding day. This suggests that the development of alternative measures may be needed to assess cognitive fluctuations subjectively over shorter time spans in heterogeneous LBD patient groups.

The additional analyses conducted in the present study also have important implications for LBD trial design. Firstly, we demonstrated that for the CRT task, between baseline and Day 5, the use of a reduced number of trials (10 or 20, compared to the standard 30) can still result in excellent test–retest reliability. This suggests that shorter duration tasks are feasible, which would reduce participant burden whilst maintaining reliability. Future work should examine if this applies to all commonly-used reaction time measures, and if this is affected by participant heterogeneity (e.g. medication use, dementia severity). The present study also provides indicative minimum sample sizes to account for the test–retest reliability of attentional outcome measures, where comparable improvements in reaction time are expected. For instance, in pharmacological studies, CRT improvements of 100-200 ms have been observed in a trial of memantine, and PoA improvements of approximately 300 ms in a trial of rivastigmine, relative to placebo, both at 24 weeks [15, 33]. Therefore, these results can directly inform the design of future LBD clinical trials.

Overall, these findings should be replicated in a larger sample size, where this is the primary aim of the study, to confirm the test–retest reliability of these attentional measures. Future studies should ensure that they have sufficient statistical power to be able to examine reliability at follow-up stages, as although we have demonstrated that demanding clinical trials, with daily assessments, are feasible in an LBD population [19], in the present study, a relatively large number of participants (approximately 20% of participants at 12 weeks) were lost to follow-up, although this consistent with other LBD trials [e.g. 30]. Finally, future studies should also examine whether the attentional measures which were used in the present study could be further optimised. Whilst it was beyond the scope of the present study, future work should assess the utility of attentional testing in the measurement of cognitive fluctuations. Whilst difficult to define, cognitive fluctuations are believed to consist of a cognitive component, which is apparent in the DLB-specific attentional and working memory dysfunction, and an arousal component, which can influence attention [7, 34–37]. Therefore, studies should examine if the cognitive and arousal components independently, or additively, contribute to the symptom of cognitive fluctuations, as this may result in better objective measurement tools.

Specific strengths of the current study include the comprehensive and well-characterised nature of the patients, as the LBD diagnosis was made by two highly-experienced clinicians; additionally, participants demonstrated medication stability before entering the study. This is particularly important as reaction time measures are very sensitive to the effects of a range of pharmacological agents. A further strength of this study is in the fact that the participants included in the trial had a wide range of dementia severity. This included participants who demonstrated high impairment, thus maximising the generalisability of the results due to the participant heterogeneity.

Despite the important implications of the present study, there are several potential limitations. The main limitation is in the post hoc secondary analysis of clinical trial data; however, persuading participants to take part in an intensive observational study of this nature may be practically difficult, particularly when the participants have a range of dementia severity. Additionally, we could not compare test–retest reliability between DLB and PDD participants due to the lack of statistical power. However, both patient groups display similar levels of attentional impairment [5, 6]. Whilst there may be domain-specific differences between DLB and PDD in the rate of cognitive decline over time, it is not known if this applies to objective attention [38] and future studies may wish to assess whether this can influence attentional reliability. Additionally, although minor variations in time between baseline and Day 1 data collection may have influenced the results, given the excellent levels of reliability observed, this is likely to have had only a minimal effect and was controlled for by repeating the baseline tests where the delay was judged to be excessive.

A further limitation is that these data were obtained from participants from a trial investigating the effects of tDCS upon visual hallucinations. Although attentional measures were not the primary outcome measure in the intervention group, tDCS may affect brain networks beyond the targeted localised region of interest [39]. It is extremely unlikely that this would have affected the results of the present study, since attentional brain regions were not targeted, and several variables can affect tDCS efficacy in dementia populations [40]. Nonetheless, future prospective studies should specifically examine the reliability of these measures outside of intervention studies. Additionally, due to the absence of patients without cognitive fluctuations, it was not possible to specifically compare the reliability between patients with, and without, cognitive fluctuations. Although our results suggest that cognitive fluctuation severity may not influence the reliability of reaction time measures, with the exception of the composite Cognitive Reaction Time, at least in the short-term and when patients are divided on the basis of whether fluctuations are, or are not, clinically-significant, further work is needed to confirm this. Finally, due to data non-normality, we could not assess the reliability of untransformed data, with the exception of the CRT. Further studies, with larger sample sizes, should examine untransformed data reliability, since most LBD trials report untransformed data [24]. However, in the context of LBD trials, the transformation of data does not present any technical challenges.

In conclusion, several computerised attentional measures show excellent levels of test–retest reliability. The reciprocal composite Power of Attention measure displays the highest test–retest reliability, both short-term, and up to 12 weeks follow-up.

## Supplementary Information

Below is the link to the electronic supplementary material.

Supplementary file1 (DOCX 15 KB)

Supplementary file2 (DOCX 13 KB)

Supplementary file3 (DOCX 13 KB)

## Data Availability

The data that support the findings of this study are available from the corresponding author upon reasonable request.
