# Supplementary material for: Quantifying test-retest reliability of repeated objective attentional measures in Lewy body dementia
Source: J Neurol. 2022 Jan 27;269(7):3605–13. doi: 10.1007/s00415-022-10977-4 (PMC9217900; doi:10.1007/s00415-022-10977-4)
Supplement: Supplementary file 1 — Supplementary file1 (DOCX 15 KB) [file 415_2022_10977_MOESM1_ESM.docx]

| *Supplementary Table 1:*  Test-retest reliability of Day 0 to Day 5 attentional measures in low and high fluctuators | | | | |
| --- | --- | --- | --- | --- |
|  | Low fluctuators  (CAF scores ≤ 5; *n =* 12) | | High fluctuators  (CAF scores > 5; *n =* 24) | |
| Attentional measure | ICC | 95% CI | ICC | 95% CI |
| SRT: 1/mean RT to correct answers | 0.978^a^ | (0.947 – 0.994) | 0.871^e^ | (0.762 – 0.940) |
| SRT: 1/coefficient of variation (%) | 0.722^a^ | (0.352 – 0.919) | 0.637^e^ | (0.326 – 0.833) |
| CRT: 1/mean RT to correct answers | 0.978^b^ | (0.950 – 0.993) | 0.966^f^ | (0.937 – 0.985) |
| CRT: 1/coefficient of variation (%) | 0.824^b^ | (0.602 – 0.945) | 0.824^f^ | (0.673 – 0.920) |
| DV: 1/mean RT, correct answers | 0.961^a^ | (0.906 – 0.989) | 0.948^f^ | (0.903 – 0.977) |
| DV: 1/coefficient of variation (%) | 0.779^a^ | (0.486 – 0.935) | 0.813^g^ | (0.649 – 0.917) |
| 1/PoA | 0.995^c^ | (0.987 – 0.999) | 0.968^g^ | (0.939 – 0.986) |
| CogRT | -0.110^d^ | (-2.241 – 0.729) | 0.504^f^ | (0.071 – 0.777) |
| ^a^ (*n* = 10); ^b^ (*n* = 11) ^c^ (*n* = 8); ^d^ (*n* = 9); ^e^ (*n* = 21); ^f^ (*n* = 20); ^g^(*n* = 19)  CAF: Clinical Assessment of Fluctuations scale | | | | |
