# Supplementary material for: Quantifying test-retest reliability of repeated objective attentional measures in Lewy body dementia
Source: J Neurol. 2022 Jan 27;269(7):3605–13. doi: 10.1007/s00415-022-10977-4 (PMC9217900; doi:10.1007/s00415-022-10977-4)
Supplement: Supplementary file 2 — Supplementary file2 (DOCX 13 KB) [file 415_2022_10977_MOESM2_ESM.docx]

| *Supplementary Table 2:*  Test-retest reliability of Day 0 and Day 5 questionnaire measures | | |
| --- | --- | --- |
| Cognitive measure | ICC | 95% CI |
| MMSE^a^ | 0.932 | (0.823 - 0.969) |
| CAMCOG (total)^b^ | 0.938 | (0.878 – 0.969) |
| CAMCOG (memory subscale)^b^ | 0.884 | (0.772 – 0.941) |
| CAMCOG (executive function subscale)^b^ | 0.846 | (0.696 – 0.922) |
| CAF ^a^ | 0.223 | (-0.235 – 0.550) |
| ODFAS ^a^ | 0.543 | (0.133 – 0.763) |
| ^a^ (*n* =36); ^b^ (*n* = 35) |  |  |
