# Supplementary material for: Quantifying test-retest reliability of repeated objective attentional measures in Lewy body dementia
Source: J Neurol. 2022 Jan 27;269(7):3605–13. doi: 10.1007/s00415-022-10977-4 (PMC9217900; doi:10.1007/s00415-022-10977-4)
Supplement: Supplementary file 3 — Supplementary file3 (DOCX 13 KB) [file 415_2022_10977_MOESM3_ESM.docx]

| *Supplementary Table 3:*  Test-retest reliability of Day 0 and follow-up (4 & 12 weeks) attentional measures | | |
| --- | --- | --- |
| Attentional measure | ICC | 95% CI |
| MMSE^a^ | 0.932 | (0.871 – 0.967) |
| CAMCOG (total)^a^ | 0.970 | (0.942 – 0.985) |
| CAMCOG (memory subscale)^a^ | 0.932 | (0.870 – 0.967) |
| CAMCOG (executive function subscale)^a^ | 0.932 | (0.871 – 0.967) |
| CAF^b^ | 0.818 | (0.656 – 0.911) |
| ODFAS^a^ | 0.719 | (0.463 – 0.865) |
| ^a^ (*n* = 26); ^b^ (*n* = 27) |  |  |
